# Understanding the Role of Keratins 8 and 18 in Neoplastic Potential of Breast Cancer Derived Cell Lines

**DOI:** 10.1371/journal.pone.0053532

**Published:** 2013-01-15

**Authors:** Sapna V. Iyer, Prerana P. Dange, Hunain Alam, Sharada S. Sawant, Arvind D. Ingle, Anita M. Borges, Neelam V. Shirsat, Sorab N. Dalal, Milind M. Vaidya

**Affiliations:** 1 Advanced Centre for Treatment, Research & Education in Cancer (ACTREC), Tata Memorial Centre, Kharghar, Navi Mumbai, India; 2 Department of Histopathology, Asian Institute of Oncology, S.L. Raheja Hospital, Mahim, Mumbai, India; Wayne State University School of Medicine, United States of America

## Abstract

**Background:**

Breast cancer is a complex disease which cannot be defined merely by clinical parameters like lymph node involvement and histological grade, or by routinely used biomarkers like estrogen receptor (ER), progesterone receptor (PGR) and epidermal growth factor receptor 2 (HER2) in diagnosis and prognosis. Breast cancer originates from the epithelial cells. Keratins (K) are cytoplasmic intermediate filament proteins of epithelial cells and changes in the expression pattern of keratins have been seen during malignant transformation in the breast. Expression of the K8/18 pair is seen in the luminal cells of the breast epithelium, and its role in prognostication of breast cancer is not well understood.

**Methodology/Principal Findings:**

In this study, we have modulated K8 expression to understand the role of the K8/18 pair in three different breast epithelium derived cell lines: non-transformed MCF10A, transformed but poorly invasive MDA MB 468 and highly invasive MDA MB 435. The up-regulation of K8 in the invasive MDA MB 435 cell line resulted in a significant decrease in proliferation, motility, *in-vitro* invasion, tumor volume and lung metastasis. The down-regulation of K8 in MDA MB 468 resulted in a significant increase in transformation potential, motility and invasion *in-vitro*, while MCF10A did not show any changes in cell transformation assays.

**Conclusions/Significance:**

These results indicate the role of K8/18 in modulating invasion in breast cancer -its presence correlating with less invasive phenotype and absence correlating with highly invasive, dedifferentiated phenotype. These data may have important implications for prognostication of breast cancer.

## Introduction

Breast cancer is the most prevalent form of cancer in women worldwide accounting for 23% of the total cancer cases and ranks second overall (10.9% of all cancers). It is the second most common cancer affecting women in India [1], [2]. Breast cancers have been classified into many sub-types based on their receptor status and develop from epithelial cells in the breast tissue [3]. The two major epithelial cell types found in the breast tissue are basal and luminal epithelial cells [4]. In the bilaminar breast epithelium, the basal or myoepithelial compartment represents the proliferating compartment, while the luminal compartment consists of differentiating cells [5]. The basal and luminal cell types can be distinguished based on their keratin (K) profile.

Keratins, the largest family of intermediate filament proteins are epithelia predominant in their expression [6], [7]. The filaments are formed by non-covalent coiled coil interaction between one type I acidic (K9-K28) and one type II basic (K1–K8 and K71–K74) keratins which are obligatory heteropolymers [8]. They form cytoplasmic scaffold that emanates from the plasma membrane and spreads throughout the cytoplasm to provide shape and rigidity to the cells [9]. Apart from their structural role, they also provide a platform for various signalling events and form a complex with multiple proteins involved in signalling networks that regulate functions like cell cycle progression, apoptosis, cellular response to stress, cell size, protein synthesis and membrane trafficking [10], [11], [12], [13].

An important feature of keratins is that their expression is regulated by tissue type and also during differentiation [11], [14], [15], [16], [17]. Stratified epithelia express different keratin pairs in different compartments. The cells of the basal compartment express the keratin pair of K5/14. As the basal cells differentiate they express K4/13 in the internal epithelium and K1/10 in the cornified epithelium [6], [18], [19], [20], [21]. In breast epithelium the basal/myoepithelial cells (proliferation compartment) express K5 and K14, while the luminal cells (differentiation compartment) express K8 and K18 [5], [6]. The strictly regulated tissue and differentiation specific pattern of expression is suggestive of the fact that the keratins may have tissue specific functions.

The keratin pair of K8 and K18 is the first pair to be expressed in embryogenesis, and expression of this pair is restricted later to simple (e.g. liver, pancreas, kidney etc.) [6], [12] and mixed (e.g. breast, lungs etc.) epithelia [6], [21], [22]. This pair is known to execute various regulatory functions, which include modulation of protein localization, protein targeting/trafficking and apoptosis [10]. Further, over-expression of K8/18 is observed in adenocarcinomas [6], [23]. Aberrant expression of this pair is found in squamous cell carcinomas (SCC) irrespective of their site of origin [24], [25], [26], [27], [28], [29]. Previous studies in our laboratory and others have shown aberrant expression of K8/18 contributes to malignant transformation of stratified epithelial cells [30], [31]. Recent studies from our laboratory have shown that K8/18 pair mediates neoplastic progression via α6β4 integrin mediated signalling in oral cancer derived cell line [32] and that K8 is required for the transformation induced by loss of plakophilin3 (PKP3) in simple epithelia [33].

There are differing reports about the role of K8/18 pair in breast cancer progression. K8/18 when expressed along with vimentin have been associated with drug resistance, invasion and metastasis in breast cell carcinomas and melanomas [34], [35], [36], [37]. Other reports have suggested that the elevation of K18 expression in breast cancer patients correlates with favourable prognosis [38] and that the loss of K8 in conjunction with aberrant expression of vimentin correlates with early metastasis and poor prognosis [39], [40]. Over-expression of K18 in MDA MB 231 breast carcinoma cells, which are invasive and highly metastatic, produced differentiated phenotype [5]. Thus, the role of K8/18 in breast cancer progression is not yet clear.

In this communication we have made an attempt to decipher the role of K8/18 in neoplastic progression of mixed epithelia like breast. We have over-expressed K8 in an invasive breast cancer cell line (MDA MB 435) and down-regulated K8 expression in a non-transformed (MCF10A) and transformed less invasive breast cancer cell line (MDA MB 468). Our results show that over-expression of K8 in an invasive breast cancer cell line resulted in significant decrease in motility, *in-vitro* invasion and metastasis *in-vivo*. Down-regulation of K8 in non-transformed cell line did not result in any phenotypic changes but in a transformed less invasive cell line down-regulation of K8 resulted in increase in anchorage independence, significant increase in motility and invasion *in-vitro*. These results indicate that K8 may modulate motility and invasion in breast cancer progression.

## Materials and Methods

### Ethics Statement

All the protocols for animal studies were reviewed and approved by Institutional Animal Ethics Committee (IAEC) of ACTREC (Approval ID: 09/2008). IAEC is constituted under the guidelines of the Committee for the Purpose of Control and Supervision of Experiments on Animals (CPCSEA), Government of India.

### Cell Culture

The MDA MB 435 breast cancer cell line, derived from the pleural effusion of a 51-years old woman with breast carcinoma [41] was procured from Dr. Lalitha Samant from South Alabama University, USA. The cells were cultured in Dulbecco’s Modified Eagle’s Medium (DMEM; Gibco, Invitrogen, Carlsbad, CA): Ham’s F12 Gibco (1∶1), and 10% fetal bovin serum (FBS; Hyclone Thermo Scientific, Lafayette, CO) and standard antibiotic mixture containing penicillin, streptomycin and amphotericin B. The doubling time of these cells was found to be 38 hours. The MDA MB 468 breast cancer cell line, isolated from pleural effusion of a 51- years old woman with metastatic adenocarcinoma of the breast [41] was procured from Dr. Sushant Kacchyap from Johns Hopkins University, USA. The cells were cultured in DMEM, supplemented with 10% FBS and standard antibiotic mixture containing penicillin, streptomycin and amphotericin B. The doubling time of MDA MB 468 cells was found to be 48.5 hours. MCF10A cell line, derived from fibrocystic non-transformed, immortalized breast epithelial cells [42] was procured from Dr. Kalpana Joshi from Nicholas Piramal, Mumbai, India. The cells were cultured in DMEM:Ham’s F12 (1∶1) Gibco, 10% FBS, Supplement cocktail (10 µg/ml Insulin, 0.5 µg/ml Hydrocortisone, 20 ng/ml EGF all from Sigma-Aldrich, USA) and standard antibiotic mixture containing penicillin, streptomycin and amphotericin B. The doubling time of these cells was found to be 50 hours. All cell lines were cultured at 37°C and 5% CO_2_ atmosphere.

### Plasmids and Constructs

K8 gene cloned in pCDNA3 (Invitrogen) was used for over-expression of K8. The shRNA to K8 gene - shRNA 8.2 (previously described) [32] was used for down-regulation of K8.

### Transfections

To generate stable cell lines constitutively expressing K8, MDA MB 435 cells (P-54) were transfected using lipofectamine Plus (Invitrogen) according to the manufacture’s protocol with 2 µg of either the vector pCDNA3 alone or K8 expression vector pCDNA3-K8. The transfectants were plated in medium containing G418, (Sigma-Aldrich, USA) at a concentration of 1200 µg/ml. To determine whether K8 loss results in increased neoplastic transformation, MDA MB 468 (P-15) and MCF10A (P-9) cells were transfected using Superfect (Qiagen) and ICAfectin 441(Eurogenetic) respectively with 2 µg of either vector pTU6.PURO alone or K8 shRNA (shRNA 8.2) cloned in pTU6 puro vector. The shRNA 8.2 was previously designed [32] as follows: Four different oligo sequences against K8 gene were used to generate pTU6-PURO based shRNA construct. The constructs were validated for K8 knockdown efficiency by co-transfecting with K8-GFP in HEK293 cells. The most effective shRNA i.e. shRNA 8.2 was used further for K8 down–regulation in MDA MB 468 and MCF10A cell lines to generate stable clones. The stable transfected clones were selected in medium containing 0.4 µg/ml of puromycin (Sigma-Aldrich USA). All the positive clones were screened by assessing their K8 levels by western blotting.

### Flow Cytometry

The cells were grown for 48 hours and were harvested by trypsinization. For the assay, 1×10^6^ cells were fixed in 1% paraformaldehyde for 15 minutes at 4°C. The cells were then treated with FACS buffer (PBS with 1%FBS, 0.02% Sodium azide and 0.1% triton X-100) for 5 minutes. The cells further incubated with anti- K8 (M20 clone) monoclonal antibody for 45 minutes at 4°C. After incubation with primary antibody, cells were washed three times with FACS buffer. The cells were then incubated with Alexa-Fluor-488-conjugated anti-mouse-IgG secondary antibody (Molecular Probes) for 45 minutes at 4°C. Cells were then analysed with FACS Calibur (Becton Dickinson, San Jose, CA) flow cytometer. The mean fluorescence intensity was measured in arbitrary units using Msiy Cell Quest software.

### Antibodies

The following antibodies were used:K8 (mouse monoclonal, clone M20; working dilution 1∶8000; K18 (mouse monoclonal, clone CY-90; working dilution 1∶8000), β-actin (mouse monoclonal, clone AC-74; working dilution 1∶8000) all from Sigma-Aldrich, USA, E-Cadherin (mouse monoclonal, working dilution 1∶5000), from BD Transduction Lab, β-4 integrin (rabbit polyclonal, clone H-101 working dilution 1∶1000) from Santa Cruz Biotechnology, K7 (mouse monoclonal RCK105, working dilution 1∶1000) from Thermo Scientific, Pierce UK), secondary antibody HRP-conjugated anti-mouse, anti-rabbit (working dilution 1∶8000; GE Healthcare) For immunofluorescence experiments, the vimentin antibody (clone 13.2) Sigma Aldrich, USA and the keratin antibodies listed above were used at a dilution of 1∶200. The secondary antibody, Fluorosceinisothiocyanate (FITC)-conjugated sheep anti–mouse IgG (F6257, Sigma-Aldrich, USA), was used at a dilution of 1∶200. For flow cytometry secondary antibody Alexa flour 488 anti-mouse-IgG secondary antibody (A1101 Molecular Probes) was used.

### Keratin Extraction and Western Blotting

The keratins were extracted in high salt extraction buffer and processed as described previously [43]. Whole-cell lysates were prepared in SDS lysis buffer (2% SDS, 50 mM Tris-HCl, pH 6.8, 0.1% BME (β-Mercaptoethanol), and 10% glycerol. A protease inhibitor cocktail (Calbiochem, San Diego, CA) was added to lysis buffer. Equal amount of protein was loaded and resolved on SDS–PAGE gels followed by Western blotting. The signals were detected using ECL Plus detection system (Amersham) according to the manufacturer’s protocol on X-ray films (Carestream, Kodak). The exposed X-ray films were processed in Promax X-ray film processor.

### Real-time PCR

Real time PCR analysis was carried out to determine the K8 and K18 levels for MDA MB 435 clones. A total of 2 µg of RNA was reverse transcribed using pdN_6_ random primers. Real time PCR analysis was performed on 10 ng of cDNA using gene specific primers for *K8* (Forward primer-5′ AGATGAACCGGAACATCAGC-3′, Reverse primer- 5′TCCAGCAGCTTCCTGTAGGT-3′), for *K18* (Forward primer: 5′-TGAGACGTACAGTCCAGTCCTT-3′, Reverse primer-5.’ GCTCCATCTGTAGGGCGTAG-3′) SYBR green based real-time quantitative PCR was performed on the ABI 7900HT Fast Real Time PCR System (Applied Biosystems, Foster, CA, USA**).** The relative gene expression was quantified by comparative Ct method using *GAPDH* (Forward primer -5′-TCAACGACCACTTTGTCAAGC-3′, Reverse primer-5′- TACTTTATTGATGGTACATGACAAGG - 3′) as house keeping control.

Plasmid copy number of all the clones was determined by real time PCR analysis on the genomic DNA isolated from the clones and parental cells. To determine the plasmid copy number of K8 up-regulated clones, real time PCR was performed for neomycin resistant gene (Forward primer: 5′-CGTTGGCTACCCGTGATATT-3′ and Reverse primer 5′-AAGAAGGCGATAGAAGGCGA-3′), and for K8 knockdown clones, real time PCR was performed for puromycin resistant gene (Forward-5′ CGCAGCAACAGATGGAAGGC-3′ Reverse primer: 5′-CCGCTCGTAGAAGGGGAGGT-3′). GAPDH primer recognizing genomic *GAPDH* gene (Forward primer: 5′ AGGGTCTACATGGCAACTG-3′ and Reverse primer: 5′- CGACCACTTTGTCAAGCTCA-3′) was used as reference to determine the copy number.

### Mass Spectrometry Analysis

Keratins extracted using high salt buffer were run on SDS-PAGE. The bands from Coomassie stained gels were excised for the mass spectrometry analysis. Briefly, for the analysis, the gel pieces were washed with water and after thoroughly rinsing, the gel pieces were gradually dehydrated using 50 mM NH_4_HCO_3_:Acetonitrile (1∶1) for 15 minutes and then with 100% Acetonitrile. The gel pieces were then vacuum dried and further incubated with 10 mM Dithiotreitol at 56°C for reduction and with 55 mM Iodoacetamide in dark at room temperature for alkylation. The gel pieces were then washed with 1∶1 ratio of NH_4_HCO_3_: Acetonitrile. The final wash with 100% Acetonitrile was given to dehydrate the gel pieces were vacuum dried followed by an overnight digestion with 10 ng/µl trypsin in 25 mM NH_4_HCO_3_ After digestion, the peptides were extracted using 50% Acetonitrile and 5% TFA (Tri fluoro acetic acid) solution. The tryptic protein digests were reconstituted using 10% Acetonitrile and 0.1% TFA solvent before subjecting to mass spectrometry analysis. A reconstituted sample and Matrix α-CyanoHydroxyCinnamic acid (CHCA) (1∶1) were mixed properly and were loaded onto the ground steel MALDI plate and subjected to mass spectrometry (MS) analysis on Ultraflex-II MALDI TOF-TOF (Brucker Daltonics) machine.

### Immunofluorescence and Laser Confocal Microscopy

For immunofluorescence cells were grown on coverslips for 48 hours and treated with 0.03% Triton X-100 in chilled methanol for 90 seconds and fixed in chilled methanol for 10 minutes at −20°C. The fixed cells were washed in PBS twice and incubated with respective primary antibodies at room temperature for 1 hour. The cells were further washed with PBS four times each for 10 minutes and incubated with the secondary antibodies at room temperature for 1 hour. Confocal images were obtained with a LSM 510 Meta Carl Zeiss Confocal system with an Argon 488 nm laser. All images were obtained using an Axio Observer Z.1 microscope numerical aperture (NA 1.4) at a magnification of 63X with 2X optical zoom.

### Lipid Droplets (Nile Red Staining)

Cells were stained with Nile red for lipid droplets (marker of cell differentiation). Briefly, the stock solution of Nile red 1 mg/ml in acetone was diluted in PBS (1∶1000). The fixed cells (4% paraformaldehyde) were incubated with diluted Nile red for 5 minutes at room temperature, rinsed with PBS and observed for the presence of lipid droplets by confocal microscopy. Confocal images were obtained with a LSM 510 Meta Carl Zeiss Confocal system with Helium/Neon 543 nm lasers. All images were obtained using an Axio Observer Z.1 microscope numerical aperture (NA 1.4) at a magnification of 63X with 2X optical zoom.

### Cell Proliferation Assay

Cells were seeded, in triplicate in a 96-well microtiter plate in 100 µl complete medium. Proliferation was studied every 24 hours up to a period of 4 days using MTT assay as described previously [44]. A growth curve was prepared from three independent experiments by plotting O.D. at 540 nm (on *y*-axis) against time (on *x*-axis).

### Soft Agar Colony Forming Assay

Cells were tested for anchorage-independent growth using soft agar colony-forming assay as described previously [30]. The assay was repeated thrice with 3 replicates each time.

### Cell Motility

The stably transfected cells were tested for change in motility by scratch wound as well as transwell assay. For scratch wound assay the cells were grown in 35 mm plates to 95% confluency. Cells were incubated with fresh medium, containing 10 µg/ml mitomycin-C for 3 hours. After incubation, medium was discarded and wounds were scratched with the help of sterile 2 µl pipette tip. The cells were fed with fresh medium and observed under an Axiovert 200 M Inverted Carl Zeiss microscope fitted with a stage maintained at 37°C and 5% CO_2_. Cells were observed by time lapse microscopy and images were taken every 10 minutes for 20 hours using an Axio CamMRm camera with a 103 phase 1 objective. Migration was measured using Axiovision software version 4.5 (Zeiss).

For assessing motility by transwell assay 2×10^5^ cells were seeded into the top chamber with serum-free medium containing 0.1% BSA and medium containing 10% serum was added to the lower chamber of the Boyden chamber (polyvinyl pyrrolidone-free polycarbonate filter with 8-µm pore size inserts, BD Pharmingen, San Diego, CA). The cells were incubated for 16 hours. Motility of cells to the underside of the membrane was detected by wiping the upper side with cotton swab and staining the underside cells with Mayer's Hematoxylin and Eosin (H&E) solution. The cells were counted under microscope in five random fields after staining. The assay was repeated thrice with 3 replicates each time.

### *In-vitro* Invasion Assay

Matrigel invasion assay was performed to check the invasive potential of the stably transfected clones. For the assay 2×10^5^ cells were seeded into the top chamber with serum-free medium containing 0.1% BSA and medium containing 10% serum was added to the lower chamber of the Boyden chamber coated with 40 µl (1 µg/µl stock) Matrigel (coated on a polyvinyl pyrrolidone-free polycarbonate filter with 8-µm pore size inserts, BD Pharmingen, San Diego, CA). Cells that invaded through the matrix to the bottom of the filter were then fixed and stained with H&E solution. After staining, the cells were counted under microscope in five random fields. The assay was repeated thrice with 3 replicates each time.

### Tumorigenicity Assay

To determine the tumorigenic potential 1×10^6^ cells were injected in the mammary fat pad of five SCID mice. Tumor volume (mm^3^) was calculated by the formula (1/2 × width × width × length) as previously reported [45]. After 7 weeks the tumors were excised and wound was surgically sealed. The surgery was performed under anesthesia (mixture of 900 µg of ketamine hydrochloride and 160 µg of Xylazine hydrochloride in normal saline) and all possible efforts were made to minimize suffering. The animals were sacrificed after 4 weeks and the vital organs were collected for histological examination. Five µm thick sections from the formalin fixed and paraffin embedded tissue samples were stained with H&E solution and slides were viewed under light microscope.

### Statistical Analysis

Two groups of data were compared by performing a *t-*test statistical analysis p<0.05 was considered significant.

## Results

The role of K8/18 pair in breast cancer progression is not well understood and the available literature is inconsistent [4], [5], [35], [36], [38], [39]. To determine if K8/18 play a role in regulating tumor progression in breast tissue, three breast derived cell lines - MCF10A (immortalised but not transformed), MDA MB 468 (transformed but less invasive) and MDA MB 435 (transformed, highly invasive and metastatic) served as model system in this study. To determine if K8 and K18 levels in these cells correlate with the degree of transformation, the levels of K8/18 were determined in the three cell lines using Western blot and immunofluorescence analysis. K8 and K18 levels were high in MCF10A cells, present at lower levels in MDA MB 468 cells and undetectable in MDAMB 435 cells (Figure S1A and S1B). These results suggested that the levels of K8 and K18 reflect the degree of transformation and invasion in these cells, with the non-transformed cell line showing the highest and the invasive cell line showing the lowest expression**.** MDA MB 435 cells showed high expression of vimentin, while it was weak or undetectable in MDA MB 468 and MCF10A cells (Figure S1A and S2A).

**Figure 1:**
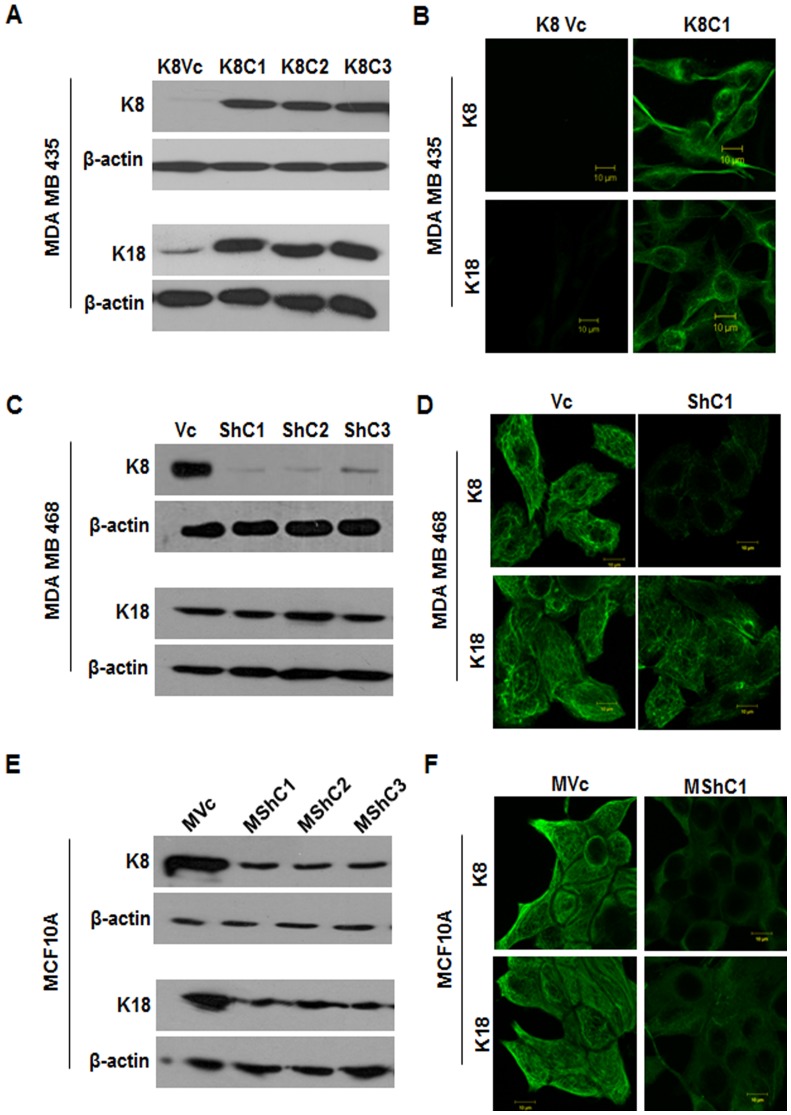
Analysis of K8 and K18 expression in K8 (over-expressed) MDA MB 435 and K8 (down-regulated) MDA MB 468 and MCF10A clones. Western blot analysis of K8/18 using mAbs to K8 and K18 respectively (**A**) MDA MB 435 K8 over-expressed (K8C1, C2 and C3) and vector control (K8Vc) clones. (**C**) MDA MB 468 K8 down-regulated (ShC1, C2 and C3) and vector control (Vc) clones. (**E**) MCF10A K8 down-regulated (MshC1, C2 and C3) and vector control (MVc) clones. β-actin was taken as loading control. Representative confocal images of K8 and K18 filaments (**B**) K8 over-expressed in MDA MB 435 (K8C1) and vector control (K8Vc) clones. (**D**) MDA MB 468 K8 down-regulated (ShC1) and vector control (Vc) clones. (**F**) MCF10A K8 down-regulated (MShC1) and vector control (MVc) clones. Scale bar: 10 µm. **Note:** Formation of K18 filaments in K8 over-expressed MDAMB 435 clones; No change in K18 filaments in K8 down-regulated MDA MB 468 clones; diffused K18 filament formation in K8 down-regulated MCF10A clones.

**Figure 2:**
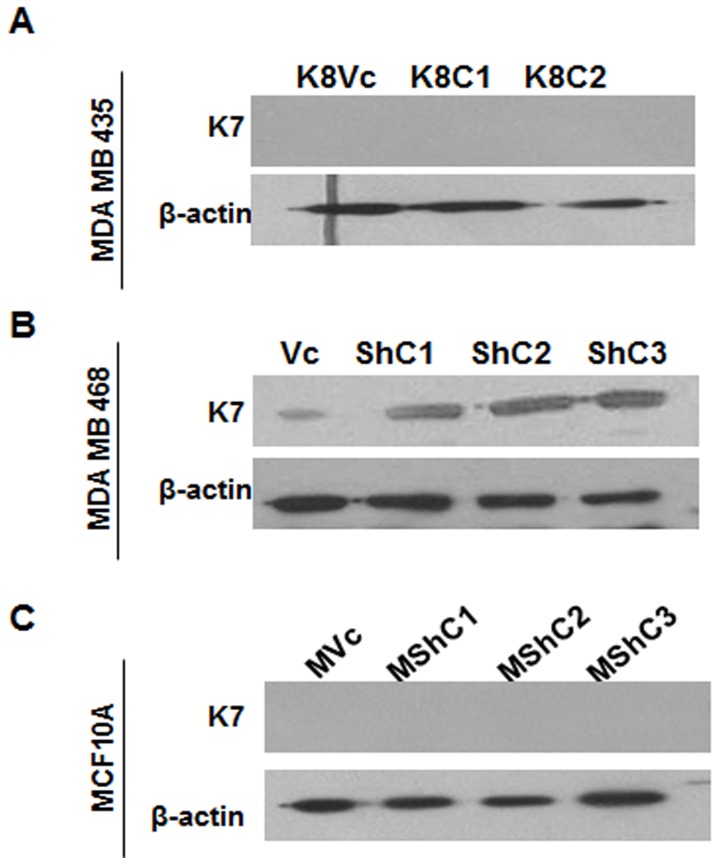
Analysis of K7 expression in K8 (over-expressed) MDA-MB-435 and K8 (down-regulated) MDA-MB-468 and MCF10A clones. Western blot analysis of K7 using mAb to K7 (**A**) MDA MB 435 K8 over-expressed (K8C1, C2 and C3) and vector control (K8Vc) clones. (**B**) MDA MB 468 K8 down-regulated (ShC1, C2 and C3) and vector control (Vc) clones. (**C**) MCF10A K8 down-regulated (MshC1, C2 and C3) and vector control (MVc) clones. β-actin was taken as loading control. **Note:** K7 up-regulation in K8 down-regulated MDA MB 468 clones. B).

**Figure 3:**
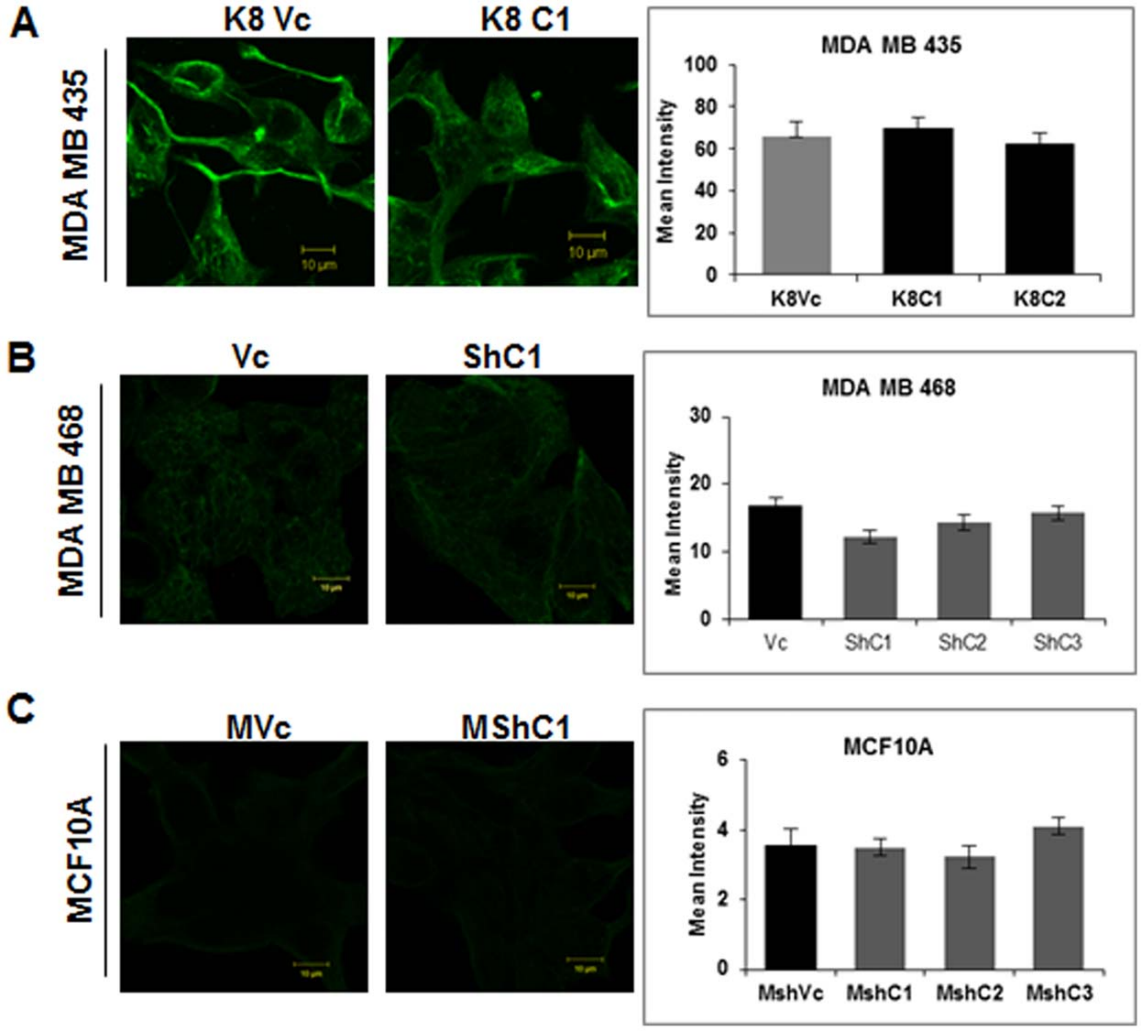
Analysis of vimentin expression in K8 (over-expressed) MDA-MB-435 and K8 (down-regulated) MDA-MB-468 and MCF10A clones. Representative confocal images of vimentin filaments (**A**) K8 over-expressed MDA MB 435 (K8C1) and vector control (K8Vc) clones. (**B**) MDA MB 468 K8 down-regulated (ShC1) and vector control (Vc) clones. (**C**) MCF10A K8 down-regulated (MshC1) and vector control (MVc) clones. Scale bar: 10 µm. For measuring the intensity of the cells the 4% laser with emission filter band pass of 505–550 was used. All the scanning conditions of gain offset and laser percentage were kept same and applied for all images of vector and their respective clones with secondary control as threshold. The mean fluorescence intensity of 20 cells was calculated and the average of three independent experiments was calculated. Results are mean of ± SE of three independent experiments performed **Note:** No change in vimentin expression or filament formation on K8 up−/down-regulation.

To determine if expression of the K8/18 pair inhibits transformation in breast epithelia, MDA MB 435 cells were transfected with either vector alone (pCDNA3) or K8 expression construct (K8- pCDNA3). Three stable clones expressing K8 (K8C1, C2 and C3) and empty vector transfected clone (K8Vc) were obtained. The clones K8C1 and C2 along with the vector control K8Vc were selected for further analysis (Figure 1A). Up-regulation of K8 in MDA MB 435 clones as compared to vector control clone and parental cells was also confirmed using flow cytometry analysis with antibody to K8 (M 20 clone, sigma) (Figure S6A). The K8 over-expressed clones showed up-regulation of its binding partner K18 (Figure 1A) as previously reported [5] without much change in their mRNA levels (Figure S7). The K8 over-expressed clones formed K8/18 filaments (Figure 1B). K7 was not detected in vector control or K8 overexpressed clones (Figure 2A). Vimentin levels remained unchanged in cells over-expressing K8 (Figure 3A). For MDA MB 435 clones the plasmid copy number was found close to 1.

To determine whether loss of K8 leads to an increase in transformation in the MCF10A or MDA MB 468 cells, a previously described shRNA construct to K8, shRNA8.2 [32] or the empty vector control pTU6-puro were transfected to generate stable clones. The levels of K8 and K18 were assessed by Western blotting. MDA MB 468 K8 knockdown clones (ShC1, C2, and C3) exhibited efficient down regulation in K8 levels (undetectable) as compared to the vector control clone (Vc) (Figure 1C). Down-regulation of K8 in MDA MB 468 clones as compared to vector control clone and parental cells was also confirmed using flow cytometric analysis with antibody to K8 (M-20 clone, Sigma) (Figure S6B). There was no change in K18 levels in K8 down-regulated clones (Figure 1C), in contrast to what has been observed previously by us and others [32], [33]. Immunofluorescence analysis showed no K8 filaments, yet K18 filaments were observed (Figure 1D) indicating that K18 formed filaments possibly by binding with some other type II keratin like K7 [46], [47]. Analysis of K8 down-regulated MDA MB 468 clones (ShC1, C2, and C3) showed up-regulation of K7 as compared to the vector control clone (Vc) (Figure 2B). Total keratin profile of K8 down-regulated MDA MB 468 clones by high salt extraction demonstrated no down-regulation in expression levels of any other keratins except K8 indicating the specificity of the shRNA used for down-regulation of K8 (Figure S4). The absence of K8 was confirmed using Western blot analysis (Figure 1C and 1D) and MS (Table S1). MCF10A stable K8 knockdown clones (MShC1,C2, C3) showed 80% down-regulation in K8 and 60% down-regulation in K18 protein levels respectively as compared to the vector control clone (MVc) (Figure 1E, S5A and S5B). Down regulation of K8 in MCF10A clones as compared to vector control clone and parental cells was also confirmed using flow cytometric analysis with antibody to K8 (M-20 clone, Sigma) (Figure S6C) The immunofluorescence analysis revealed formation of diffused filaments in the K8 knockdown MCF10A clones (MShC1, C2 and C3) as compared to the vector control clone (MVc) (Figure 1F). K7 was not detected in K8 knockdown (MShC1, C2, and C3) as well as vector control clones (MVc) (Figure 2C). The levels of vimentin remained unchanged in MDA MB 468 and MCF10A derived K8 knockdown clones (Figure 3B and 3C). Plasmid copy number was found to vary from 1–2 for MDA MB 468 and MCF10A clones.

A differentiated mammary gland exhibits a specific secretory function of producing milk. Human milk mainly is constituted of fat globules containing triglycerides. Triglycerides are neutral lipids that can be stained by Nile red [48], [49]. To determine if change in K8 levels affected differentiation, the levels of lipid droplets were measured in the K8 over-expressed and K8 knockdown clones. The K8 over-expressing MDA MB 435 clones (K8C1 and C2) did not show any significant change in lipid droplet levels as compared to the vector control clone (K8Vc) (Figure S3A). The MDA MB 468 (ShC1, C2 and C3) and MCF10A (MShC1,C2 and C3) derived K8 knockdown clones did not show much change in the lipid droplet levels as compared to the vector control clones (Vc or MVc) (Figure S3B and S3C). These results indicate that change in levels of K8 did not have any effect on the differentiation status of the cells.

**Figure 4:**
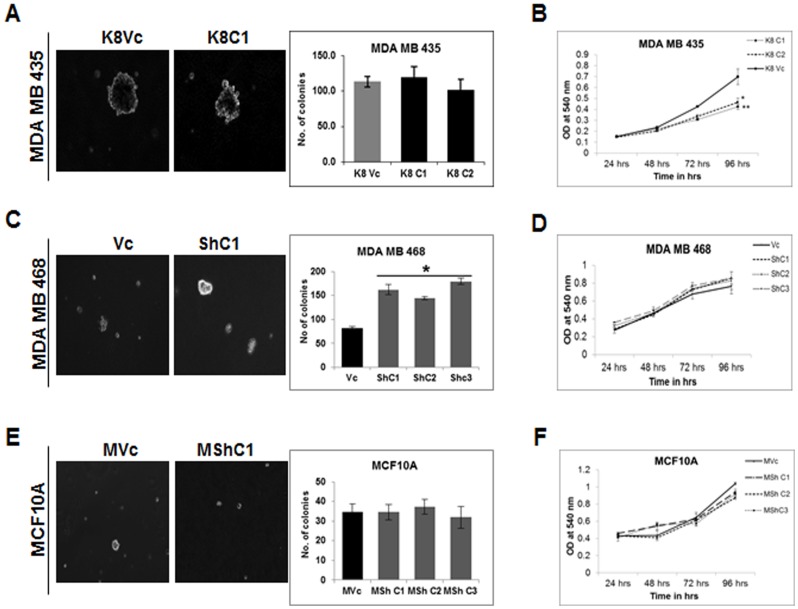
Analysis of changes in soft agar colony forming potential and cell proliferation on K8 up−/down-regulation. Representative phase contrast images (10X) of colonies formed in soft agar (**A**) MDA MB 435 K8 over-expressed (K8C1) and vector control (K8Vc) clones. Histogram showing number of colonies of MDA MB 435 K8 overexpressed (K8C1, and C2) and vector control (K8Vc) clones (right hand side). (**C**) MDA MB 468 K8 down-regulated (ShC1) and vector control (Vc) clones. Histogram showing number of colonies of MDA MB 468 K8 down-regulated (ShC1, C2and C3) and vector control (Vc) clones (right hand side). (**E**) MCF10A K8 down-regulated (MShC1) and vector control (MVc) clones. Histogram showing number of colonies of MCF10A K8 down-regulated (MshC1, C2 and C3) and vector control (MVc) clones (right hand side). **Note:** Increase in soft agar colonies formed in K8 down-regulated MDA MB 468 clones (*p<0.05 by students t-test). Cell proliferation curves of (**B**) MDA MB 435 K8 overexpressed (K8C1, C2 and C3) and vector control (K8Vc) clones. (**D**) MDA MB 468 K8 down-regulated (ShC1, C2 and C3) and vector control (Vc) clones. (**F**) MCF10A K8 down-regulated (MShC1, C2 and C3) and vector control (MVc) clones. Cell proliferation was plotted against time. Results are mean ± SE of three independent experiments performed in triplicate. **Note:** Decrease in proliferation in K8 over-expressed clones of MDA MB 435 (*p<0.05, **p<0.01 by students t-test).

In order to assess the effects of K8 expression on tumorigenesis, soft agar colony forming assays were performed. The MDA MB 435 K8 over-expressing clones (K8C1 and C2) did not show any significant change in the size and the number of the colonies formed in the soft agar as compared to the vector transfected clone (K8Vc) (Figure 4A). The K8 knockdown clones of MDA MB 468 (ShC1, C2 and C3) demonstrated a significant increase in size and number of colonies compared with vector control clone (Vc) (Figure 4C). There was no change in the number or volume of the soft agar colonies formed in MCF10A K8 knockdown clones (MShC1, C2 and C3) as compared to the vector control clone (MVc) (Figure 4E). Thus, K8 loss led to alteration in soft agar colony formation only in the MDA MB 468 cell line.

To determine if an alteration in K8 levels led to changes in proliferation, MTT assays were performed in the K8 over-expressing or K8 knockdown clones. The K8 over-expressed MDA MB 435 clones (K8C1, C2) showed significantly decreased growth as compared to the vector control clone (K8Vc) (*p<0.05, **p<0.01) (Figure 4B). The MDA MB 468 K8 knockdown clones (ShC1, C2 and C3) and MCF10A K8 knockdown clones (MShC1, C2 and C3) derived K8 knockdown clones did not demonstrate significant difference in the proliferation as compared to their respective vector control clones (Vc and MVc) respectively (Figure 4D and 4F).

**Figure 5:**
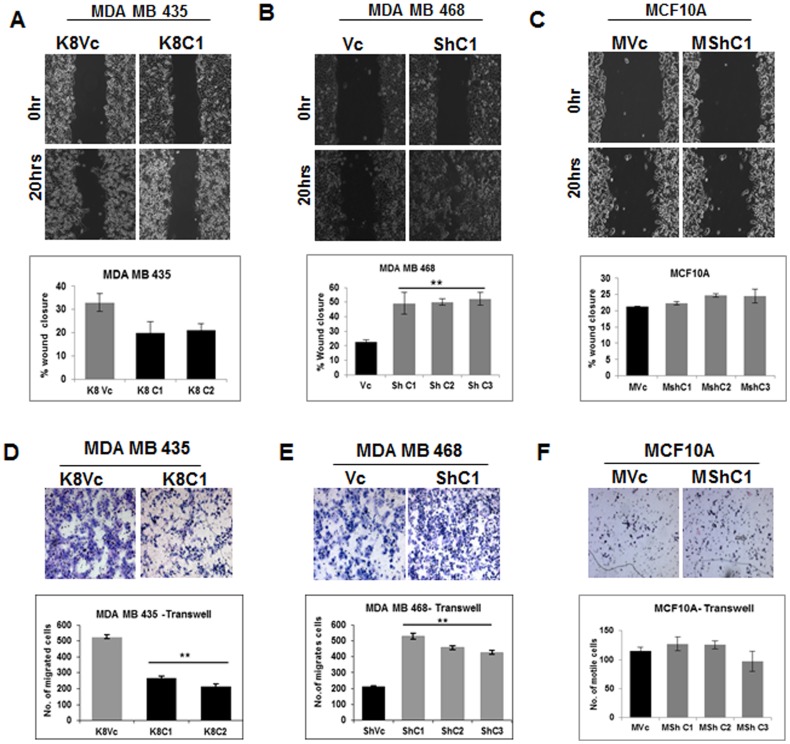
Analysis of change in motility on K8 up−/down-regulation. Representative 10X Phase contrast images of time lapse microscopy at 0 hour and 20 hours showing wound healing (**A**) MDA MB 435 K8 over-expressed (K8C1) and vector control (K8Vc) clones. Histogram showing % wound closure at the end of 20 hours of MDA MB 435 K8 over-expressed (K8C1 and C2) and vector control (K8Vc) clones (lower panel). (**B**) MDAMB 468 K8 down-regulated (ShC1) and vector control (Vc) clone. Histogram showing % wound closure at the end of 20 hours of MDA MB 468 K8 down-regulated (ShC1, C2and C3) and vector control (Vc) clones (lower panel). (**C**) MCF10A K8 down-regulated (MShC1) and vector control (MVc) clone. Histogram showing % wound closure at the end of 20 hours of MCF10A K8 down-regulated (MShC1, C2and C3) and vector control (MVc) clones (lower panel). Results are mean of ± SE of three independent experiments performed. Migration rate was calculated by AxioVision software. **Note:** Significant Increase in motility in K8 down-regulated MDA MB 468 clones as compared to vector control. Motility of K8 up−/down-regulated clones by transwell assay: Representative images of H &E stained migrated cells (**D**) MDA MB 435 K8 over-expressed (K8C1) and vector control (K8Vc) clones. Histogram showing number of migrated cells at the end of 16 hours of MDA MB 435K8 over-expressed (K8C1 and C2) and vector control (K8Vc) clones (lower panel). (**E**) MDAMB 468 K8 down-regulated (ShC1) and vector control (Vc) clone. Histogram showing number of migrated cells at the end of 16 hours of MDA MB 468 K8 knockdown clones (ShC1, C2 and C3) and vector control (Vc) clones (lower panel). (**F**) MCF10A K8 down-regulated (MShC1) and vector control (MVc) clone. Histogram showing number of migrated cells of MCF10A K8 down-regulated (MShC1, C2 and C3) and vector control (MVc) clones (lower panel). Results are mean of ± SE of three independent experiments performed **Note:** Significant decrease in motility in K8 over-expressed MDA MB 435 clones and significant increase in motility in K8 down-regulated MDA MB 468 clones (**p<0.01 by students t-test).

Previously published results from our laboratory and others have suggested that alterations in K8 expression lead to changes in motility [32], [33]. To determine whether these changes in motility would be observed in the cell systems used in this report, motility was assessed by performing wound healing assay as well as transwell assay. Decrease in motility was observed in MDA MB 435 (K8C1 and C2) derived K8 over-expressing clones. However, the decrease in motility was not statistically significant when compared to vector control clone (K8Vc) in the wound healing assay (Figure 5A), while transwell method showed significant decrease in motility in K8 over-expressed MDA MB 435 (K8 C1 and C2) clones as compared to vector control clone (K8Vc) (Figure 5D). There was a significant increase in the motility in MDA MB 468 derived K8 knockdown clones (ShC1, C2 and C3) as compared to vector control clone (Vc) in both scratch wound as well as transwell assay (Figure 5B and 5E). No change in the motility was observed in the MCF10A K8 knockdown clones (MShC1, C2 and C3) as compared to the vector control clone (MVc) in both scratch wound and transwell assay (Figure 5C and 5F). These results indicate that K8 presence impedes motility in highly invasive and transformed cell lines and its absence enhances motility in a less invasive transformed cell line.

**Figure 6:**
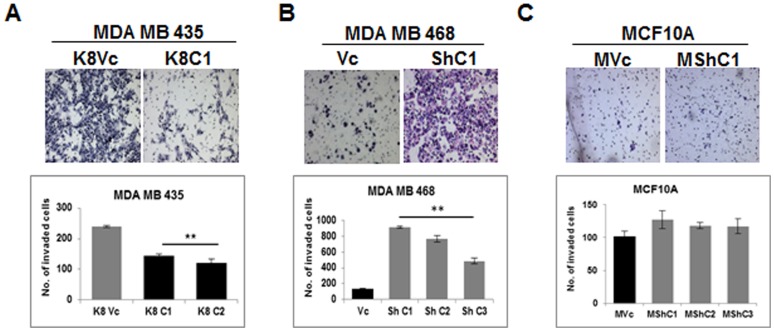
Analysis of change in *in-vitro* invasion on K8 up−/down-regulation. Representative 10X images of H&E stained membrane showing invaded cells. (**A**) MDA MB 435 K8 over-expressed (K8C1) and vector control (K8Vc) clones. Histogram showing number of invaded cells of MDA MB 435 K8 overexpressed (K8C1 and C2) and vector control (K8Vc) clones (lower panel). (**B**) MDA MB 468 K8 down-regulated (ShC1) and vector control (Vc) clones. Histogram showing number of invaded cells of MDA MB 468 K8 down-regulated (ShC1, C2 and C3) and vector control (Vc) clones (lower panel). (**C**) K8 down-regulated MCF10A (MShC1) and vector control (MVc) clones. Histogram showing number of invaded cells of MCF10A K8 down-regulated (MShC1, C2 and C3) and vector control (MVc) clones (lower panel). Results are mean of ± SE of three independent experiments performed **Note:** Decreased invasion in K8 over-expressed MDA MB 435 clones (K8C1 and C2) and increased invasion in K8 down-regulated MDA MB 468 clones (ShC1, C2, and C3) as compared to their respective vector controls (K8Vc) and (Vc).

Our previous results have demonstrated that changes in K8 expression lead to changes in metastatic progression [32], [33]. To determine if changes in K8 expression led to changes in invasion, matrigel invasion assays were performed. The K8 overexpressing MDA MB 435 clones (K8C1 and C2) exhibited significant reduction in invasion as compared to vector control clone (K8Vc) (**p value <0.01) (Figure 6A). There was significant increase in invasion in the MDA MB 468 derived K8 knockdown clones of (ShC1, C2 and C3) as compared to the vector control clone (Vc) (Figure 6B). There was no change in invasion in MCF10A derived K8 knockdown clones (MShC1, C2 and C3) as compared to vector control clone (MVc) (Figure 6C). The results indicate that K8 up-regulation in invasive cell line led to decrease in invasion while K8 down-regulation in less invasive cell line led to an invasive phenotype.

**Figure 7:**
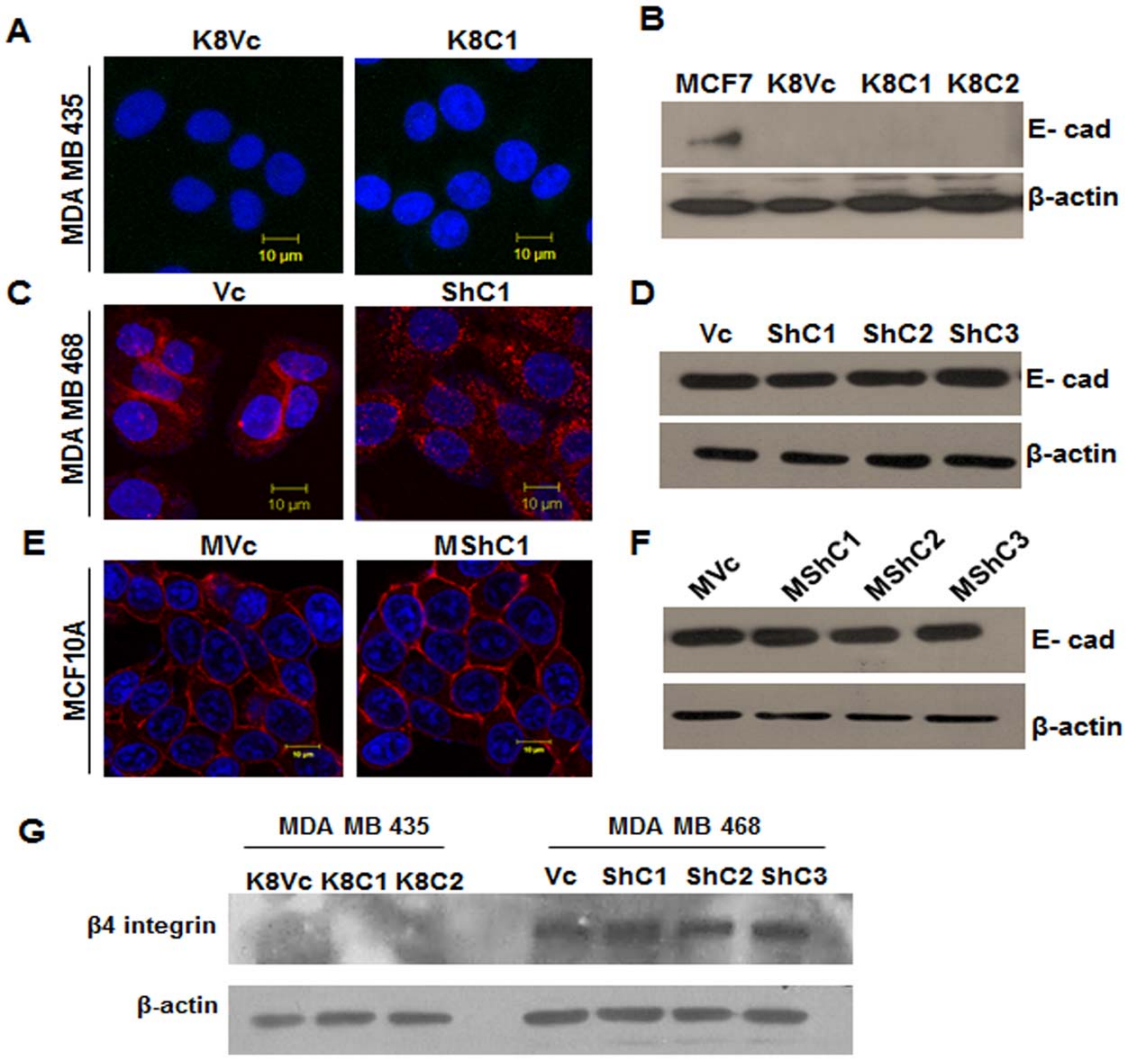
Analysis of E-cadherin expression and β4 expression on K8 up−/down-regulation. E-cadherin expression: Representative confocal images of E-cadherin in (**A**) K8 over-expressed MDA MB 435 (K8C1) and vector control (K8Vc) clones. (**C**) MDA MB 468 K8 down-regulated (ShC1) and vector control (Vc) clones. (**E**) MCF10A K8 down-regulated (MC1) and vector control (MVc) clones. Scale bar: 10 µm. Note: Cytoplasmic localization of E-cadherin in K8 down-regulated MDA MB 468 clones, Nuclei (blue) is stained with DAPI. Western blot analysis of E-cadherin: (**B**) MDA MB 435 K8 over-expressed (K8C1, C2 and C3) and vector control (K8Vc) clones. MCF7 served as positive control. (**D**) MDA MB 468 K8 down-regulated (ShC1, C2 and C3) and vector control (Vc) clones. (**F**) MCF10A K8 down-regulated (MshC1, C2 and C3) and vector control (MVc) clones. β-actin was used as loading control. **Note:** No change in E-cadherin levels on K8 up−/down-regulation. (**G**) Western blot analysis of β4 integrin: in MDA MB 435 K8 over-expressed (K8C1, C2 and C3) and vector control (K8Vc) clones and MDA MB 468 K8 down-regulated (ShC1, C2 and C3) and vector control (Vc) clones. β-actin was taken as loading control. **Note:** No change in levels of β4 integrin.

Changes in cell adhesion molecule like E-cadherin have been reported earlier in breast cancer [50]. To determine whether the change in motility and invasion was accompanied with change in E-cadherin, we analysed E-cadherin expression using Western blot and immunofluorescence analysis. K8 over-expressed MDA MB 435 clones did not show up-regulation of E-cadherin (Figure 7A and 7B). Change in localization of E-cadherin from membrane to cytoplasm was seen in K8 down-regulated clones of MDA MB 468 (ShC1, C2 and C3) while the vector control clone (Vc) showed membrane staining (Figure 7C). The Western blot analysis showed that there was no change in the levels of E-cadherin in these clones as compared to vector control clone (Figure 7D). MCF10A K8 down-regulated clones did not demonstrate any change in localization or levels of E-cadherin (Figure 7E and 7F).

Previous reports from our laboratory showed the involvement of β4 integrin mediated pathway on K8 down-regulation in human oral SCC derived cell lines [32]. To asses if the same pathway is involved in the change in motility observed in the system used in this report, we determined β4 integrin levels in MDA MB 435 K8 up-regulated (K8C1and C2) and K8 down-regulated MDA MB 468 clones (ShC1, C2 and C3) as compared to their respective vector control clones (K8Vc and Vc). We did not see any significant change in the levels of β4 integrin in MDA MB 468 clones on K8 down- regulation while in MDA MB 435 clones levels of β4 integrin remained undetected (Figure 7G).

**Figure 8:**
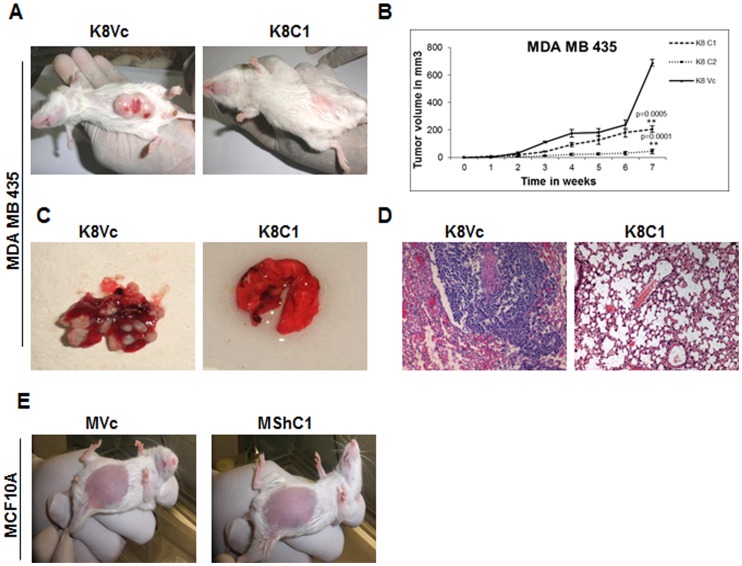
Analysis of change in tumorigenicity and metastatic potential on K8 up−/down- regulated clones of MDA MB 435 and MCF10A cell lines. (**A**) Representative images of NOD-SCID mice bearing tumors of vector control (K8Vc) and K8 over-expressed (K8C1) clones, 7 weeks after the injection in mammary fat pad. (**B**) Tumor growth was plotted against time (*p<0.05, **p<0.01 by student’s t-test). Results are mean of ± SE for five animals injected for each clone. (**C**) Representative images of excised lungs of animals injected with vector control (K8Vc) and K8 over-expressed (K8C1) clones. (**D**) H&E stained lung sections of animals injected with MDA MB 435 vector control clone (K8Vc) showing metastatic foci throughout the lungs and K8 over-expressed clone (K8C1) showing no metastasis. (**E**) Representative images of NOD-SCID mice injected with MCF10A vector control (MVc) and K8 down-regulated (MShC1) clones, 7 weeks after the injection in mammary fat pad. **Note:** Lungs of animals injected with MDA MB 435 vector control clone showing metastatic nodules and lungs of animals injected with K8 over-expressed MDAMB 435 clone (K8C1) showing no visible metastatic nodules.

To determine if the changes in migration and invasion described above led to changes in the ability to form tumors *in-vivo,* MDA MB 435 derived K8 over-expressing clones of (K8C1 and C2) and the vector control clone (K8Vc) were injected in mammary fat pad of the SCID mice. Mice injected with K8 over-expressing clones demonstrated significantly smaller tumors as compared to the vector control clone (Figure 8A and 8B) (*p<0.05, **p<0.01, by student’s t-test). The lungs of the animals, injected with K8 over-expressed clones (K8C1 and C2) showed no visible metastatic foci, while the lungs of animals injected with vector control clone (K8Vc) demonstrated visible metastatic foci throughout the lungs (Figure 8C). The histological examination of the lungs revealed large foci of metastasis which were detected throughout the lungs in animals injected with vector control clone (K8Vc) whereas two out of five animals injected with K8 transfected clone (K8C1) showed a few metastatic foci. No metastasis was observed in animals injected with clone K8C2 (Figure 8D). NOD-SCID mice injected with MCF10A K8 down-regulated (MShC1, C2 and C3) and vector control (MVc) clones did not form any tumors (Figure 8E).

In summary, overexpression of K8 in MDA MB 435 resulted in decrease in proliferation and invasion *in-vitro* and decrease in metastasis *in-vivo*. Inhibition of K8 expression in MDA MB 468 resulted in an increase in cellular transformation and invasion *in-vitro*. The down-regulation of K8 in MCF10A did not show any phenotypic alterations *in-vitro* as well as *in-vivo*. These results suggest that K8 may function as a suppressor of transformation and metastasis in breast epithelia in contrast to results obtained from other cell type [30], [32], [33].

## Discussion

The results presented in this report show that K8/18 expression inversely correlates with motility, invasion *in-vitro* and metastasis *in-vivo* in breast cancer cell lines. Previous reports from both our laboratory and others have shown that an increase in K8/K18 expression in squamous cell carcinomas as well as adenocarcinomas is associated with the metastatic phenotype [32], [33]. The available literature related to K8/18 expression in breast cancer progression is inconsistent. Some of the previous reports on tumor samples and cell lines suggested that the expression of K8/18 was associated with invasion and poor prognosis [4], [35], [36]. Other reports from breast tumor samples suggested that loss of K8/18 expression was associated with poor prognosis [38], [39], [40] which correlated with the results of our present study and with data previously reported for K18 expression *in-vitro* [5].

The decrease in motility, tumor size, invasion *in-vitro* and less or no metastasis in lungs of the animals injected with K8 over-expressed MDA MB 435 clones suggest that K8 may inhibit metastatic progression. K8 over-expression in MDA MB 435 was accompanied with up-regulation in K18. This may be the result of stabilization of K18 protein, as we did not observe any significant change in K18 mRNA levels on K8 up-regulation (Figure S7B). It is possible that the K8/18 filaments formed after K8 up-regulation may have led to increased rigidity and hence become less invasive [5]. In another set of experiments inhibition of K8 in a less invasive cell line (MDA MB 468) resulted in increased motility and invasion *in-vitro*. Down-regulation of K8 in MDA MB 468 did not show concomitant decrease in K18 expression and K18 filaments were still observed. The filament formation of K18 indicated possible compensatory pairing of K18 with some other type II keratin like K7 [46], [47]. This possibility was explored by analysing the total keratin profile of the cells, which showed the presence of K7, K18 and K19 in K8 down-regulated MDA MB 468 clones (Table S1). Keratin pair of K5/14 is often associated with poor prognosis in breast cancer [51] but K5 or K14 were not detected in MDA MB 468 clones even after K8 down-regulation. Western blot analysis showed up-regulation of K7 in these clones. Keratin 7 is one of the type II keratins that is expressed in breast epithelium but the expression is generally low and variable. It has been shown previously that K7 replaces K8 in its absence [46], [47]. It is possible that K18 is forming filaments with K7 in the absence of K8, although these filaments may not have the same function as that of K8/18 filaments. The invasion data suggest that loss of K8 in these cells was sufficient to increase invasion. No change in K18 filament formation suggests that in addition to the rigidity imparted by keratin filaments other functions of K8 might be important in preventing neoplastic transformation in breast tissue. We did not detect K7 in MDA MB 435 and MCF10A clones and we observe the concomitant up−/down-regulation of K18 in response to K8 up−/down-regulation, indicating that there was no compensation by K7 in these cells. In summary, these findings together underline the importance of K8/18 filaments in maintaining the non-transformed phenotype in breast cancer derived cell lines.

Previous reports have shown the role of vimentin in breast cancer progression [4], [5], [35], [36], [38], [40]. Though vimentin is widely studied in breast cancer, present study shows that change in motility or invasion were independent of vimentin expression. Results from our previous work on human oral tumor derived cell line have also indicated that the changes seen on modulation of K8 were independent of changes in vimentin expression, whose levels remained unchanged. This indicates that K8/18 and vimentin may regulate invasion and motility via independent pathways in different cell types.

Another interesting finding of our study was change in localization of E-cadherin in K8 down-regulated clones of MDA MB 468. Invasion being a multistep process and requires co-ordinated loss of cell adhesion molecules which would assist in loosening the cellular architecture and hence imparting invasiveness to the cells by helping the cells detach more easily [52]. We show here that the down-regulation of K8 in MDA MB 468 cells led to change in localization of E-cadherin from membrane to cytoplasm, without any change in protein levels. The direct link between keratins and E-cadherin is not known. Keratins form the cellular meshwork via anchoring to the desmosomal plaque. Plakoglobin (PG) is one of the desmosomal complex proteins which also forms part of adherens junction [53]. Recruitment of PKP3 by E-cadherin and PG is essential for desmosome formation [54]. Thus loss of keratin 8 in these cells may have resulted in the loss of membranous E-cadherin due to indirect effect of altered interaction of keratins with desmosomal proteins. E-cadherin being a cell adhesion molecule, its loss at the membrane might promote tumor cell dissemination. The clinical significance of the cytoplasmic expression of E-cadherin has been shown in gastric cancer where it correlated with poor prognosis [55]. Thus, the cytoplasmic localization of E-cadherin in K8 down-regulated MDA MB 468 cells may be the downstream effect of the series of alterations that may influence invasion. On the other hand, MDA MB 435 cells did not express E-cadherin and K8 up-regulation in these cells did not induce its expression. The decreased invasion in the MDA MB 435 derived K8 over-expressing clones seems to be independent of E-cadherin expression. This observation also highlights the fact that K8 might modulate invasion by diverse pathways in different breast cancer derived cell lines. To summarize, modulation of K8 expression and its effect on K8/18 filament formation does not induce or repress the levels of E-cadherin but results in re-localization of this protein. Re-localization could be the result of altered interaction of E-cadherin with other junctional complex proteins.

Previous work from our laboratory has shown that the keratin pair of K8/18 plays a positive role in tumor progression in an oral SCC derived cell line. This change is brought about by β4 integrin mediated signalling and alterations in β4 integrin levels explain most of the phenotypes observed upon K8 knockdown, including decrease in invasion, migration and tumorigenicity [32]. There are no alterations in the levels of β4 integrin on K8 up−/down- regulation in the cell lines used in this study. β4 integrin was undetected in MDA MB 435 clones whereas no change in its expression was seen in K8 down-regulated clones of MDA MB 468 as compared to the vector control clone. The role of α6β4 integrin in tumor progression is still obscure. Several reports have indicated that over-expression of this pair is seen in SCC of lung, skin, oral cavity and cervix. While its expression is down-regulated in adenocarcinomas of breast and prostate [56]. The present study indicates that the changes in motility or invasion in these breast cancer derived cell lines may not be regulated by β4 integrin mediated pathway.

The role of other integrins in breast cancer has been reported in literature. Integrins β1 and β3 are the most common type of integrins that have been shown to mediate tumor progression in breast cancer. K8/18 have been shown to mediate β1 integrin mediated cell adhesion in hepatomas [57]. Most of the previous studies show role of α3β1 integrin in promoting tumor progression and metastasis in breast cancer [58]. While its over-expression in skin carcinoma is shown to decrease the rate of carcinoma formation [59]. In several malignancies up-regulation of αvβ3 integrin correlated with tumor progression for e.g. in melanoma, glioma, ovarian cancer and breast cancer [60]. It will be interesting to see if the change in invasive property of the cell lines used in our study is brought about by any of these integrin pairs or by some other signalling pathways.

Although keratins show high tissue specificity in their expression, alterations in their expression profile have been reported in various malignancies. Previous reports from our laboratory and others have shown aberrant expression of K8/18 in precancerous lesions and SCC of oral mucosa [24], [25], [61]. We further showed that this keratin pair contributes in acquisition of transformed and metastatic phenotype of oral squamous epithelium derived cell line [30]. K8/18 expression has been shown to correlate with poor prognosis in SCC of oral cavity [28]. In breast epithelium K8/18 are normally expressed in the luminal/differentiation compartment and this study shows that loss of same keratin pair in breast cancer derived cell lines resulted in an invasive phenotype. These findings suggest the possibility that the same keratin pair may have dissimilar role in neoplastic progression of different epithelia. Hence it will be important to analyse breast tumors for K8/18 expression to understand their clinical significance. Our results of K8 down-regulation in MCF10A suggest that K8 may not be involved in changing the motility or invasive potential of a non-transformed cell lines. Hence it is tempting to speculate that K8 loss in breast cancer might be an important event in metastatic progression.

The exact mechanism by which these changes are brought about is still not understood. To understand the underlying mechanism, further detailed analysis of changes at the molecular and proteomic level in response to modulation of K8/18 level is required. This information could prove useful in developing therapeutic targets for treatment of breast carcinomas in future. It will be also important to study K8 expression in breast tumors and correlate the same with the clinical parameters of the patients so as to understand its significance in prognostication of breast cancer.

## Supporting Information

Figure S1 Analysis of K8, K18 and vimentin in MCF10A, MDA MB 468 and MDA MB 435 cell lines. Representative confocal images of K8, K18 and vimentin filaments formed in **(A)** MCF10A, MDA MB 468 and MDA MB 435 cells using mAb to K8, K18 and vimentin respectively. **(B)** Western blot analysis of K8 and K18 in MCF10A, MDA MB 468 and MDA MB 435 cells using mAb to K8 and K18 respectively. β-actin was taken as loading control. Histogram showing relative intensity of K8 and K18 of the cell lines MCF10A, MDA MB 468 and MDA MB 435 (Right hand side). The fold change was calculated using Image J software. To determine the fold intensity, Intensity of the band was normalized using β-actin as internal control. Relative intensity was measured by considering band intensity of MCF10A for K8 and K18 as 1. MDAMB 468 showed 2 fold less K8 and K18 as compared to MCF10A while in MDA MB 435 K8 expression was negligible and K18 expression was 5 fold lesser than MCF10A. **Note:** Decrease in K8, K18 expression from MCF10A to MDA MB 435 cells.
(TIF)

Figure S2 Analysis of soft agar colony forming potential in MCF10A, MDAMB 468 and MDA MB 435 cell lines. Representative phase contrast images (10X) of colonies formed in soft agar per plate by **(A)** MCF10A, MDA MB 468 and MDA MB 435. **(B)** Histogram showing number of colonies formed in soft agar. **(C)** Histogram showing volume of colonies formed in soft agar. Size of the colonies was determined using Axiovision software (*p<0.05 by student’s t-test). Results are mean of ± SE of three independent experiments performed. **Note:** Increase in number and volume of soft agar colonies formed from non-transformed MCF10A to invasive MDA MB 435 cell lines.
(TIF)

Figure S3 Analysis of differentiation status of K8 up−/down-regulated clones by lipid droplets staining using Nile red. Representative confocal images of lipid droplets staining **(A)** MDA MB 435 K8 over-expressed (K8C1) and vector control (K8Vc) clones. Histogram showing the mean intensity of MDA MB 435 K8 over-expressed (K8C1 and C2) and vector control (K8Vc) clones (right hand side). **(B)** MDA MB 468 K8 down-regulated (ShC1) and vector control (Vc) clones. Histogram showing the mean intensity of MDA MB 468 K8 down-regulated (ShC1, C2 and C3) and vector control (Vc) clones (right hand side). (**C)** MCF10A K8 down-regulated (MShC1) and vector control (MVc) clones. Histogram showing the mean intensity MCF10A K8 down-regulated (MShC1, C2 and C3) and vector control (MVc) clones (right hand side). Scale bars: 10 µm. All the scanning conditions of gain offset and laser percentage were kept same and applied for all images of vector and their respective clones with secondary control as threshold. The mean fluorescence intensity (± SE) of lipid droplets was calculated per cell by measuring fluorescence intensity of 20 cells of each experiment (using LSM10 software; Carl Zeiss MicroImaging GmbH, Jena, Germany).This was repeated thrice Results are mean of ± SE of three independent experiments performed. **Note:** No change in lipid droplet staining intensity in any of the clones on K8 up−/down-regulation.
(TIF)

Figure S4 High salt Keratin extraction. The keratin profile of MDA MB 468 K8 down-regulated (ShC1 and C2) and vector control (Vc) clones after high-salt extraction. The arrow indicates position of K8 band on the gel at molecular weight (M.W. ∼52 kDa). The numbers indicated on the left hand side indicates the gel pieces taken for the mass spectrometry analysis**. Note:** Keratin 8 was observed to be down-regulated in the K8 knockdown clones as compared to vector control.
(TIF)

Figure S5 MCF10A K8/18 down-regulation. **(A)** Histogram showing % protein expression (± S.E.) for three independent experiments of K8 in MCF10A K8 down-regulated (MShC1, C2 and C3) and vector control (MVc) clones.**(B)** Histogram showing % protein expression (± S.E.) for three independent experiments of K18 in MCF10A K8 down-regulated (MShC1, C2 and C3) and vector control (MVc) clones. The percentage of protein expression was determined by Image J software. The intensity of the K8 or K18 expression was normalized with β-actin. Vector control clone (Mvc) intensity was considered as 100% expression. **Note:** ∼60% down-regulation in K18 levels on K8 down-regulation.
(TIF)

Figure S6 Analysis of K8 expression by flow cytometry in K8 up-regulated clones of MDA MB 435, K8 down-regulated clones of MDA MB 468 and MCF10A. Histograms showing mean fluorescence intensity of K8 (± S.E.) for three independent experiments (lower panel); in **(A)** K8 up-regulated MDA MB 435 clones (K8C1 and C2) as compared to parental MDA MB 435 and vector control clone (K8Vc) **(B)** K8-down-regulated MDA MB 468 clones (ShC1, C2 and C3) as compared to parental MDA MB 468 and vector control clone (Vc). **(C)** K8 down-regulated MCF10A clones (MShC1, C2 and C3) as compared to parental MCf10A and vector control clone (Mvc) as analysed by flow cytometry.
(TIF)

Figure S7 Real time PCR analysis of K8 and K18 expression in K8 up-regulated MDA MB 435 clones. Real time PCR analysis of genes encoding **(A)** K8 gene in MDAMB 435 K8 over-expressed (K8C1, C2) and vector control (K8Vc) clones and **(B)** K18 gene in K8 over-expressed MDA MB 435 clones (K8C1 and C2) as compared to vector control clone (K8Vc) using *GAPDH* as internal control. Results are mean of ± SE of three independent experiments performed. **Note:** No significant difference in K18 levels on K8 up-regulation.
(TIF)

Table S1 Details of the bands with keratin identity.
(DOCX)
