# Supplementary figures and images for: Understanding the Role of Keratins 8 and 18 in Neoplastic Potential of Breast Cancer Derived Cell Lines
Source: PLoS One. 2013 Jan 15;8(1):e53532. doi: 10.1371/journal.pone.0053532 (PMC3546083; doi:10.1371/journal.pone.0053532)

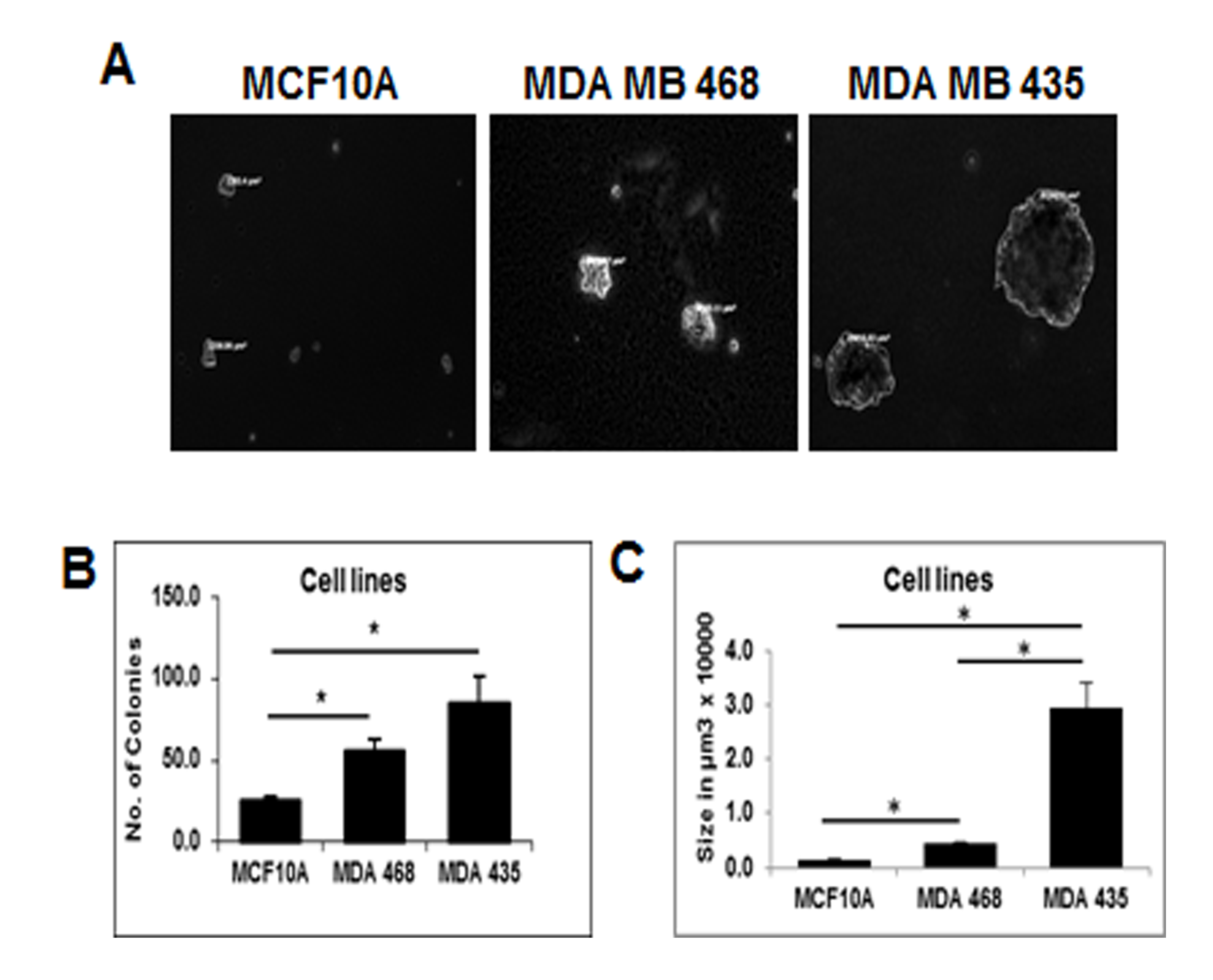

Supplement: Figure S2 — Analysis of soft agar colony forming potential in MCF10A, MDAMB 468 and MDA MB 435 cell lines. Representative phase contrast images (10X) of colonies formed in soft agar per plate by (A) MCF10A, MDA MB 468 and MDA MB 435. (B) Histogram showing number of colonies formed in soft agar. (C) Histogram showing volume of colonies formed in soft agar. Size of the colonies was determined using Axiovision software (*p<0.05 by student’s t-test). Results are mean of ± SE of three independent experiments performed. Note: Increase in number and volume of soft agar colonies formed from non-transformed MCF10A to invasive MDA MB 435 cell lines. (TIF) [file pone.0053532.s002.tif]

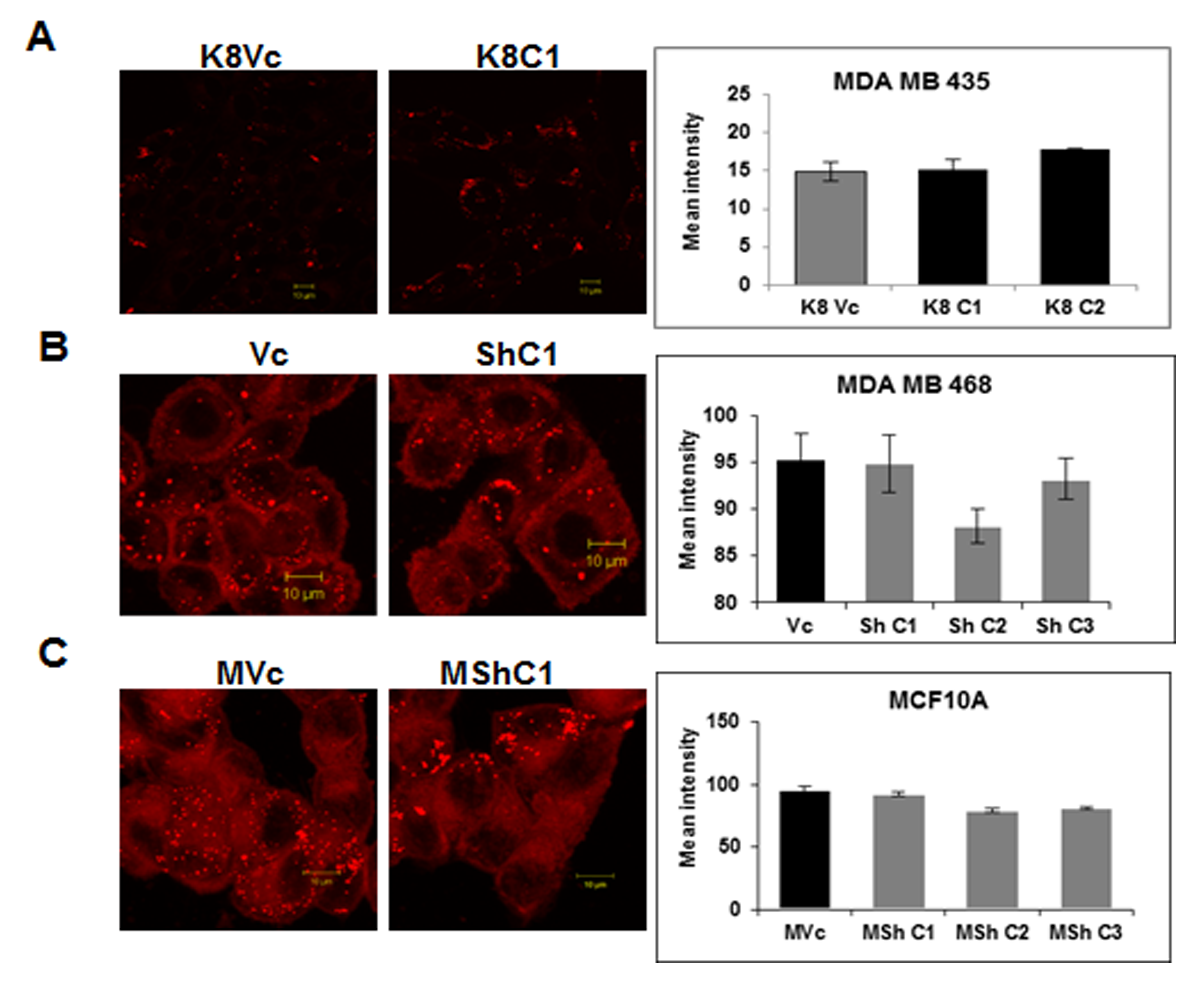

Supplement: Figure S3 — Analysis of differentiation status of K8 up−/down-regulated clones by lipid droplets staining using Nile red. Representative confocal images of lipid droplets staining (A) MDA MB 435 K8 over-expressed (K8C1) and vector control (K8Vc) clones. Histogram showing the mean intensity of MDA MB 435 K8 over-expressed (K8C1 and C2) and vector control (K8Vc) clones (right hand side). (B) MDA MB 468 K8 down-regulated (ShC1) and vector control (Vc) clones. Histogram showing the mean intensity of MDA MB 468 K8 down-regulated (ShC1, C2 and C3) and vector control (Vc) clones (right hand side). (C) MCF10A K8 down-regulated (MShC1) and vector control (MVc) clones. Histogram showing the mean intensity MCF10A K8 down-regulated (MShC1, C2 and C3) and vector control (MVc) clones (right hand side). Scale bars: 10 µm. All the scanning conditions of gain offset and laser percentage were kept same and applied for all images of vector and their respective clones with secondary control as threshold. The mean fluorescence intensity (± SE) of lipid droplets was calculated per cell by measuring fluorescence intensity of 20 cells of each experiment (using LSM10 software; Carl Zeiss MicroImaging GmbH, Jena, Germany).This was repeated thrice Results are mean of ± SE of three independent experiments performed. Note: No change in lipid droplet staining intensity in any of the clones on K8 up−/down-regulation. (TIF) [file pone.0053532.s003.tif]

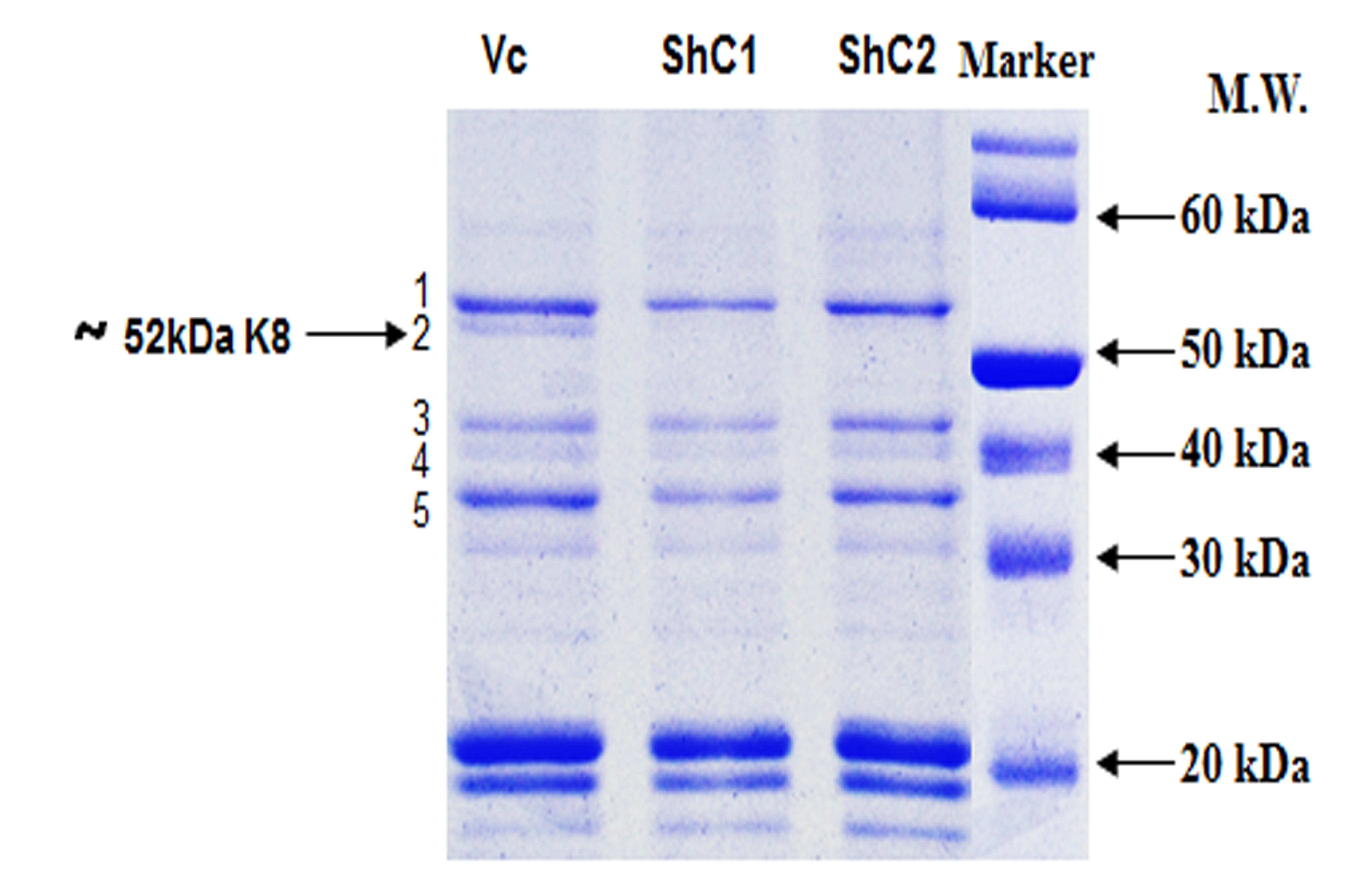

Supplement: Figure S4 — High salt Keratin extraction. The keratin profile of MDA MB 468 K8 down-regulated (ShC1 and C2) and vector control (Vc) clones after high-salt extraction. The arrow indicates position of K8 band on the gel at molecular weight (M.W. ∼52 kDa). The numbers indicated on the left hand side indicates the gel pieces taken for the mass spectrometry analysis. Note: Keratin 8 was observed to be down-regulated in the K8 knockdown clones as compared to vector control. (TIF) [file pone.0053532.s004.tif]

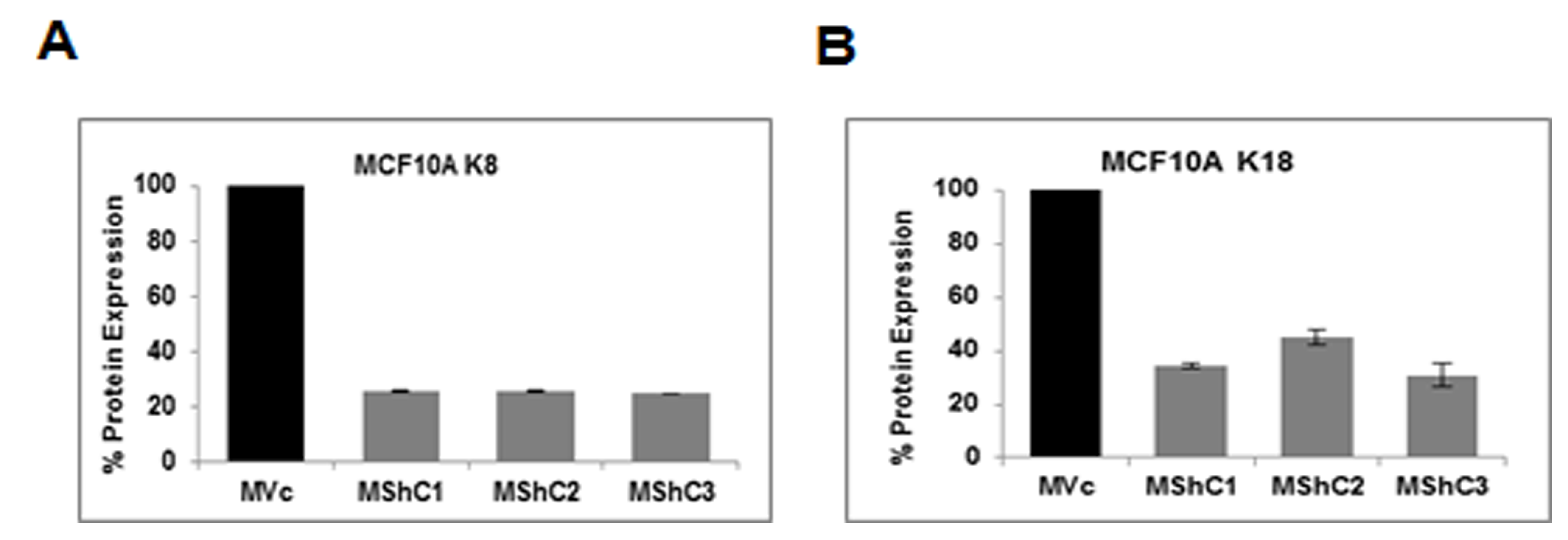

Supplement: Figure S5 — MCF10A K8/18 down-regulation. (A) Histogram showing % protein expression (± S.E.) for three independent experiments of K8 in MCF10A K8 down-regulated (MShC1, C2 and C3) and vector control (MVc) clones.(B) Histogram showing % protein expression (± S.E.) for three independent experiments of K18 in MCF10A K8 down-regulated (MShC1, C2 and C3) and vector control (MVc) clones. The percentage of protein expression was determined by Image J software. The intensity of the K8 or K18 expression was normalized with β-actin. Vector control clone (Mvc) intensity was considered as 100% expression. Note: ∼60% down-regulation in K18 levels on K8 down-regulation. (TIF) [file pone.0053532.s005.tif]

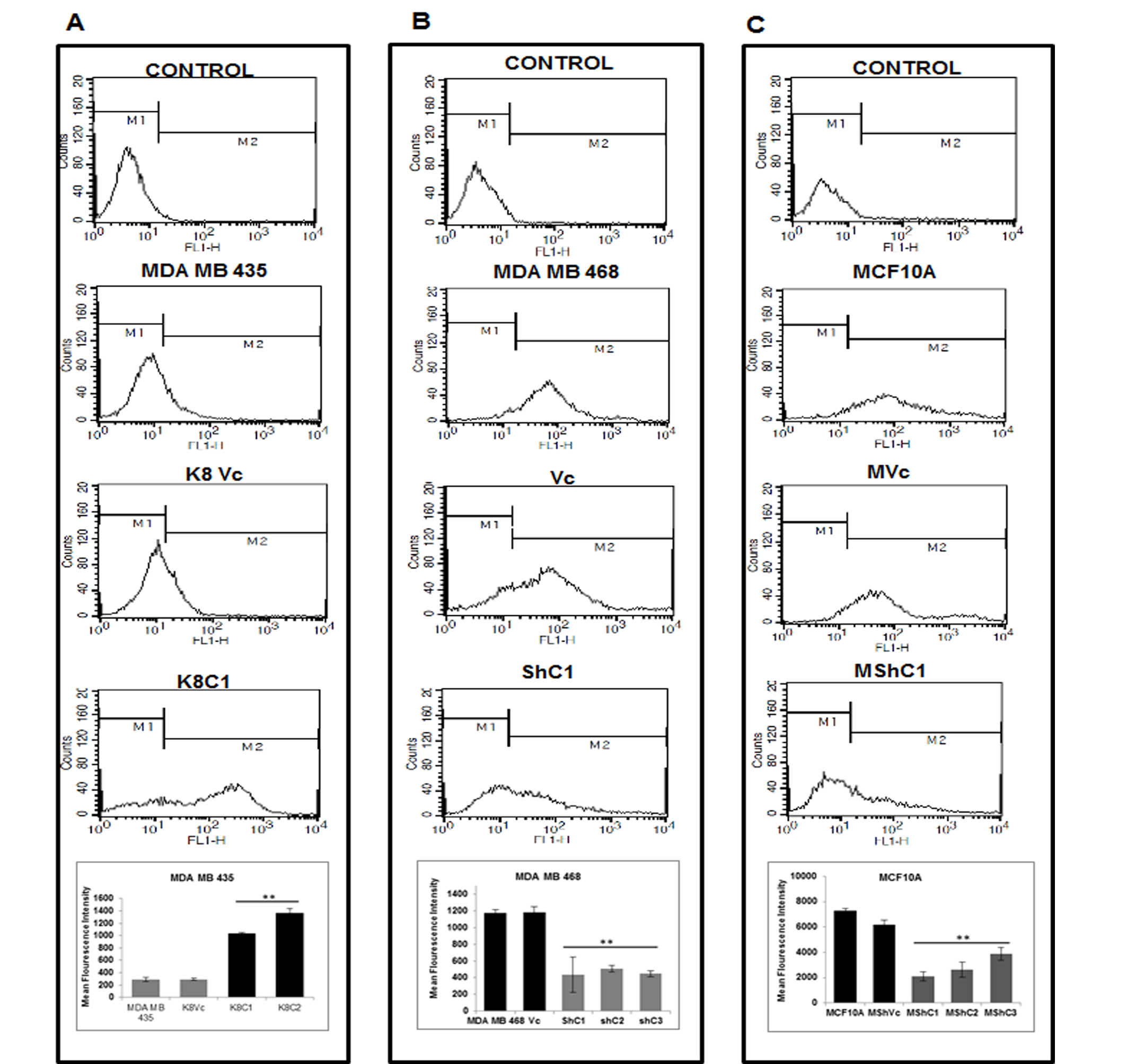

Supplement: Figure S6 — Analysis of K8 expression by flow cytometry in K8 up-regulated clones of MDA MB 435, K8 down-regulated clones of MDA MB 468 and MCF10A. Histograms showing mean fluorescence intensity of K8 (± S.E.) for three independent experiments (lower panel); in (A) K8 up-regulated MDA MB 435 clones (K8C1 and C2) as compared to parental MDA MB 435 and vector control clone (K8Vc) (B) K8-down-regulated MDA MB 468 clones (ShC1, C2 and C3) as compared to parental MDA MB 468 and vector control clone (Vc). (C) K8 down-regulated MCF10A clones (MShC1, C2 and C3) as compared to parental MCf10A and vector control clone (Mvc) as analysed by flow cytometry. (TIF) [file pone.0053532.s006.tif]

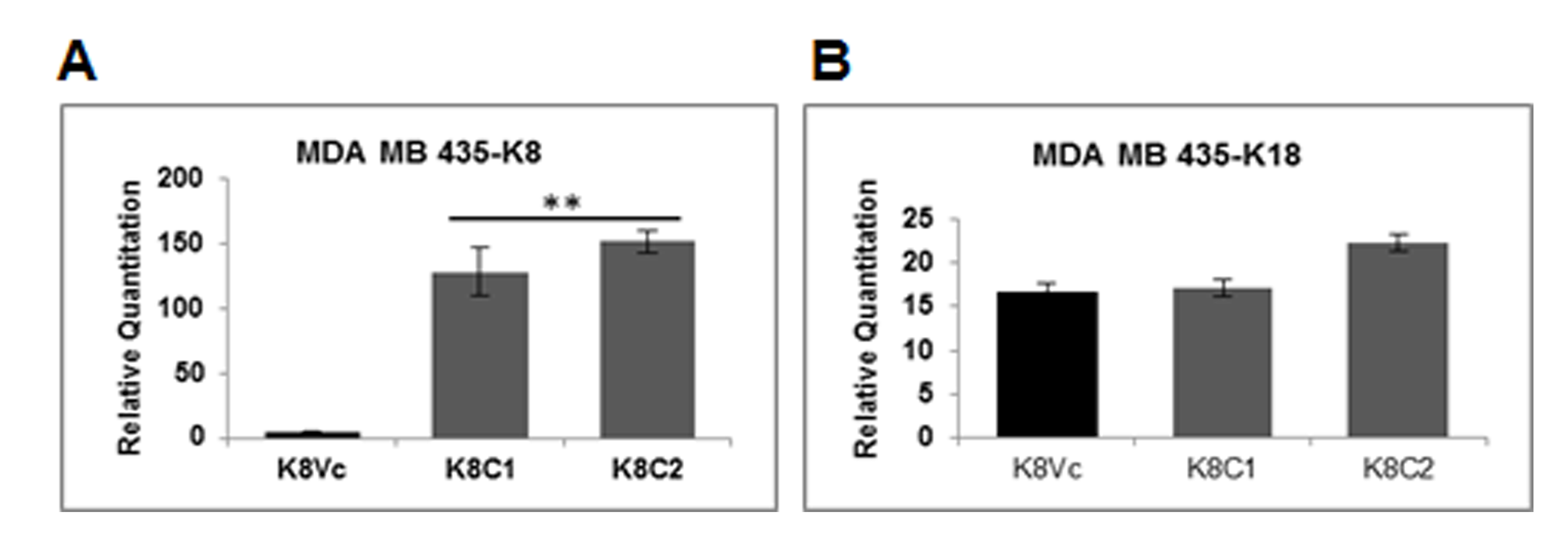

Supplement: Figure S7 — Real time PCR analysis of K8 and K18 expression in K8 up-regulated MDA MB 435 clones. Real time PCR analysis of genes encoding (A) K8 gene in MDAMB 435 K8 over-expressed (K8C1, C2) and vector control (K8Vc) clones and (B) K18 gene in K8 over-expressed MDA MB 435 clones (K8C1 and C2) as compared to vector control clone (K8Vc) using GAPDH as internal control. Results are mean of ± SE of three independent experiments performed. Note: No significant difference in K18 levels on K8 up-regulation. (TIF) [file pone.0053532.s007.tif]
