# Supplementary material for: Understanding the Role of Keratins 8 and 18 in Neoplastic Potential of Breast Cancer Derived Cell Lines
Source: PLoS One. 2013 Jan 15;8(1):e53532. doi: 10.1371/journal.pone.0053532 (PMC3546083; doi:10.1371/journal.pone.0053532)
Supplement: Table S1 — Details of the bands with keratin identity. (DOCX) [file pone.0053532.s008.docx]

**Table S1. Details of the bands with keratin identity.**

| Gel Piece No. | Protein Name | Protein Id | Accession No. | PMF | | | | | | |
| --- | --- | --- | --- | --- | --- | --- | --- | --- | --- | --- |
|  |  |  |  | Total score | Mass (Da) | pI | IC (%) | SC (%) | Total peaks | Matches |
| Lane 1: MDA MB 468 VECTOR (Vc) | | | | | | | | | | |
| Vc1 | Keratin, type II cytoskeletal 7 | K2C7_HUMAN | [P08729](http://www.uniprot.org/uniprot/P08729) | 79 | 51411 | 5.40 | 77.8 | 24.5 | 30 | 9 |
| Vc2 | Keratin, type II cytoskeletal 7 | K2C7_HUMAN | [P08729](http://www.uniprot.org/uniprot/P08729) | 97 | 51411 | 5.40 | 79.3 | 28.6 | 38 | 11 |
|  | Keratin, type II cytoskeletal 8 | [K2C8_HUMAN](http://www.matrixscience.com/cgi/protein_view.pl?file=..%2Fdata%2F20121008%2FFtGeSbuee.dat&hit=2) | [P05787](http://www.uniprot.org/uniprot/P05787) | 85 | 53671 | 5.52 | 69.3 | 28 | 38 | 11 |
| Vc3 | Keratin, type I cytoskeletal 18 | [K1C18_HUMAN](http://www.matrixscience.com/cgi/protein_view.pl?file=..%2Fdata%2F20121008%2FFtGeiGsat.dat&hit=1) | [P05783](http://www.uniprot.org/uniprot/P05783) | 194 | 48029 | 5.34 | 50 | 46.7 | 68 | 22 |
| V5 | Keratin, type I cytoskeletal 19 | K1C19_HUMAN | [P08727](http://www.uniprot.org/uniprot/P08727) | 211 | 44079 | 5.04 | 88.5 | 45 | 29 | 17 |
| Lane 2: MDA MB 468 K8 knock-down clone shC1 | | | | | | | | | | |
| shC1-1 | Keratin, type II cytoskeletal 7 | K2C7_HUMAN | [P08729](http://www.uniprot.org/uniprot/P08729) | 40 | 51411 | 5.40 | 62.9 | 10 | 25 | 5 |
| Shc1-2 | Keratin, type II cytoskeletal 7 | K2C7_HUMAN | [P08729](http://www.uniprot.org/uniprot/P08729) | 126 | 51411 | 5.40 | 80.7 | 32.6 | 31 | 12 |
| ShC1-3 | Keratin, type I cytoskeletal 18 | [K1C18_HUMAN](http://www.matrixscience.com/cgi/protein_view.pl?file=..%2Fdata%2F20121008%2FFtGeiGsat.dat&hit=1) | [P05783](http://www.uniprot.org/uniprot/P05783) | 75 | 48029 | 5.34 | 37.5 | 33.5 | 24 | 7 |
| ShC1-5 | Keratin, type I cytoskeletal 19 | K1C19_HUMAN | [P08727](http://www.uniprot.org/uniprot/P08727) | 168 | 44079 | 5.04 | 72.8 | 41.5 | 34 | 15 |
| Lane 3: MDA MB 468 K8 knock-down clone shC2 | | | | | | | | | | |
| shC2-1 | Keratin, type II cytoskeletal 7 | K2C7_HUMAN | [P08729](http://www.uniprot.org/uniprot/P08729) | 158 | 51411 | 5.40 | 88.8 | 32.6 | 26 | 13 |
| ShC2-2 | Keratin, type II cytoskeletal 7 | K2C7_HUMAN | [P08729](http://www.uniprot.org/uniprot/P08729) | 55 | 51411 | 5.40 | 51.6 | 17.7 | 20 | 6 |
| ShC2-3 | Keratin, type I cytoskeletal 18 | [K1C18_HUMAN](http://www.matrixscience.com/cgi/protein_view.pl?file=..%2Fdata%2F20121008%2FFtGeiGsat.dat&hit=1) | [P05783](http://www.uniprot.org/uniprot/P05783) | 85 | 48029 | 5.34 | 49.5 | 28.8 | 26 | 9 |
| shC2-5 | Keratin, type I cytoskeletal 19 | K1C19_HUMAN | [P08727](http://www.uniprot.org/uniprot/P08727) | 147 | 44079 | 5.04 | 75.1 | 32.8 | 27 | 13 |

Abbreviations: SC-Sequence coverage, IC –Intensity Coverage, PMF- Peptide Mass Fingerprinting, pI- Isoelectric point
